# Neutrophil extracellular trap (NET) formation characterises stable and exacerbated COPD and correlates with airflow limitation

**DOI:** 10.1186/s12931-015-0221-7

**Published:** 2015-05-22

**Authors:** Fikreta Grabcanovic-Musija, Astrid Obermayer, Walter Stoiber, Wolf-Dietrich Krautgartner, Peter Steinbacher, Nicole Winterberg, Arne Cornelius Bathke, Michaela Klappacher, Michael Studnicka

**Affiliations:** University Clinic of Pneumology, Paracelsus Medical University, Müllner Hauptstraße 48, A-5020 Salzburg, Austria; Department of Cell Biology, Biomedical Ultrastructure Research Group, University of Salzburg, Salzburg, Austria; Department of Mathematics, University of Salzburg, Salzburg, Austria

**Keywords:** COPD, Induced sputum, Neutrophils, Neutrophil extracellular traps

## Abstract

**Background:**

COPD is a progressive disease of the airways that is characterized by neutrophilic inflammation, a condition known to promote the excessive formation of neutrophil extracellular traps (NETs). The presence of large amounts of NETs has recently been demonstrated for a variety of inflammatory lung diseases including cystic fibrosis, asthma and exacerbated COPD.

**Objective:**

We test whether excessive NET generation is restricted to exacerbation of COPD or whether it also occurs during stable periods of the disease, and whether NET presence and amount correlates with the severity of airflow limitation.

**Patients, materials and methods:**

Sputum samples from four study groups were examined: COPD patients during acute exacerbation, patients with stable disease, and smoking and non-smoking controls without airflow limitation. Sputum induction followed the ECLIPSE protocol. Confocal laser microscopy (CLSM) and electron microscopy were used to analyse samples. Immunolabelling and fluorescent DNA staining were applied to trace NETs and related marker proteins. CLSM specimens served for quantitative evaluation.

**Results:**

Sputum of COPD patients is clearly characterised by NETs and NET-forming neutrophils. The presence of large amounts of NET is associated with disease severity (*p* < 0.001): over 90 % in exacerbated COPD, 45 % in stable COPD, and 25 % in smoking controls, but less than 5 % in non-smokers. Quantification of NET-covered areas in sputum preparations confirms these results.

**Conclusions:**

NET formation is not confined to exacerbation but also present in stable COPD and correlates with the severity of airflow limitation. We infer that NETs are a major contributor to chronic inflammatory and lung tissue damage in COPD.

## Background

Chronic obstructive pulmonary disease (COPD) is usually a progressive neutrophilic inflammatory airway disorder following long-term exposure to external stresses, such as inhaled tobacco smoke. Periods of stable condition alternate with severe episodes of worsening (exacerbations), leading to increasing impairment of lung function. Such lung function decline provides the basis for the classification of the disease according to international guidelines [1]. COPD is widespread and affects approximately 10 % of the adult population in westernised countries [2]. It has substantial impact on the quality of life and on life expectancy [3], currently being the third leading cause of death at the global scale [4]. COPD is characterised by recurrent bacterial infection [5, 6] along with a massive infiltration of the lung tissue by neutrophils, even affecting airway smooth muscle [7, 8]. This makes the disease a prime candidate for the involvement of neutrophil extracellular traps (NETs) in pathogenesis. NETs are web-like extracellular structures of decondensed chromatin associated with histones and enzymes such as neutrophil elastase (NE) and myeloperoxidase (MPO) that are all both antimicrobial and potentially cytotoxic. They are released by activated neutrophils, mainly in a distinct process of cell death termed NETosis [9]. Recruitment of neutrophils into NETosis is mediated by a variety of molecular signals that bind to neutrophil surface receptors, among them microbial breakdown products and the chemokine IL-8. Reaction cascades involve the generation of reactive oxygen species (ROS) by NADPH oxidase and the citrullination of histone H3 by peptidyl arginine deiminase 4 (PAD4) [10–12]. Although NETs are an important component of innate immunity, their role is ambiguous, acting between bactericidal defence and host tissue damage [13, 14]. NETs are loaded with strongly alkaline histones and degradative enzymes, and are associated with ROS release and autoantibody induction by citrullinated proteins. Thus they have a high cytotoxic potential, and evidence is accumulating that they directly contribute to host cell death and chronic tissue damage when formed in excess or are insufficiently cleared by mechanisms that are still poorly understood [15–18]. Such tissue damage is most likely for the chronic airway diseases cystic fibrosis (CF) and allergic asthma [19, 20] and may therefore also affect alveolar walls in COPD. Thus, the further clarification of the role of NETs in COPD may, in the long term, open new therapeutic perspectives. Until now, cessation of smoking has been the most effective intervention to slow down the decline in forced expiratory volume per second (FEV1) and thus the progression of the disease [21]. Routine anti-inflammatory treatment with steroids has so far been of limited success in COPD [22], and the presence of neutrophils may be even enhanced under steroid therapy [23].

In such context, we have recently provided new evidence that the sputa of patients with exacerbated COPD contain large amounts of NETs [24]. In the present study, we build upon these results by testing whether (i) excessive NET generation is restricted to the exacerbation episodes of COPD or whether it also occurs during the stable periods of the disease, and (ii) whether the abundance of NETs correlates with the degree of airflow limitation as measured by FEV1. Persons without airflow limitation with high and low noxious exposure (smokers and non-smokers, respectively) are used as controls.

## Methods

### Subjects

We examined samples of induced sputum from four groups of subjects: (i) COPD patients with acute exacerbations (n = 16), (ii) COPD patients with stable disease as seen during outpatient visits (n = 28), (iii) smoking controls (n =17), and (iv) non-smoking controls (n = 15), both of these controls without airflow limitation (Table 1). Note that portions of the sputum samples from patients with exacerbated COPD and non-smoking controls were also utilised for our previous work characterising NET micromorphology in COPD sputa as compared to *in vitro* induced NETs [24].

**Table 1:** Characteristics of study groups

Study group	Age	Sex		FEV1 % predicted	GOLD grade					Smoking history pack years		
	mean (min-max)	m	f	mean (min-max)	0	I	II	III	IV	10-40	41-70	>70
Exacerbated COPD	67.5 (46–87)	11	5	32.3 (14–57)	0	0	2	4	10	6	9	1
Stable COPD	66.5 (42–74)	15	13	48.0 (23–80)	0	1	10	8	8	9	12	7
Smoking controls	47.5 (40–57)	9	8	91.1 (80–109)	17	0	0	0	0	14	2	1
Non-smoking controls^a^	59.7 (41–77)	7	8	106.3 (93–129)	15	0	0	0	0	1	0	0

^a^Note that non-smoking controls included only one ex-smoker who stopped smoking more than 10 years before entering the study

COPD patients were recruited from outpatients and inpatients seen at the University Clinic of Pneumology in Salzburg, Austria. Smoking and non-smoking controls were recruited through announcements in local media. When entering the study, all subjects had their clinical history taken and underwent physical pulmonary examination and post-bronchodilator lung function testing according to ATS/ERS guidelines using a Jäger bodyplethysmograph. Inclusion criteria for participation in the study were: (i) age over 40 years, (ii) no history of asthma. COPD was defined on the basis of post-bronchodilator spirometry (FEV1 < 80 % predicted and FEV1/FVC < 0.7) and a reported history of current or former smoking (>10 pack-years) according to the international guidelines [1]. These guidelines were also applied to diagnose exacerbations. Inclusion criteria for controls were: no signs of COPD as determined by medical history, physical examination and lung function testing (FEV1/FVC ratio > 0.7); additionally for smoking controls: current smokers with a smoking history of minimum 10 pack-years; additionally for non-smoking controls: never smokers or no smoking for at least the last ten years. Smoking controls were on average rather younger that COPD subjects. This is because it proved impossible to find a sufficient number of older smokers with intact lung function (among over 100 candidate subjects that were screened for participation in this study, we found no smokers aged over 60 that matched the inclusion criteria). Long-term medication of all COPD subjects was left unchanged. Acutely exacerbated subjects additionally received intravenous and oral corticosteroids (dexamethasone and prednisolone, respectively), antibiotics, short acting beta-agonists and short acting anticholinergics, also according to international guidelines [1]. Smoking and non-smoking controls did not receive any medication. Note that evidence to date clearly indicates that anti-inflammatory therapy with glucocorticoids (specifically dexamethasone) has no influence on NET formation [15, 25, 26].

### Ethics statement

All participants gave written informed consent before entering the study. The study was approved by the Ethics Committee of Salzburg Province (full German name: Ethikkommission für das Bundesland Salzburg), Ref. No. 415-E/1171/12-2012.

### Sputum samples

Induced sputum was non-invasively collected according to the protocols of the ECLIPSE study [8] using an EasyNeb™ ultrasonic nebuliser. Briefly, sputum induction was performed by inhalation of hypertonic saline (3 % NaCl, three times, 7 min each). Lung function tests after each inhalation were performed with an EasyOne™ spirometer. Harvested sputa were examined for the presence of alveolar and brochiolar epithelial cells to ensure origin from the depth of the respiratory tract. Sputum samples were homogenised with 0.25 mg/ml Dithiothreitol (DTT). Depending upon subsequent use, the sputa were adhered to either poly-D-lysine coated coverslips (for immunostaining and scanning electron microscopy, 6–10 specimens from each subject in the study), or poly-D-lysine coated strips of Aclar® fluoropolymer, or Formvar-coated 200 mesh gold grids (both of these for transmission electron microscopy, usable specimens from most of the sputum samples). For comparison with DNA-free/NET-free state, one specimen on coverslip from each subject was treated with DNAse (2000 U/ml, 30 min, at room temperature). All specimens were fixed in phosphate buffered 4 % paraformaldehyde at 4 °C. All samples were assigned an anonymised code to ensure unbiased evaluation.

### Immunostaining

Immunodetection of neutrophil elastase (NE), citrullinated histone H3 (CitH3), and peptidylarginine deiminase 4 (PAD4) by confocal laser microscopy (CLSM) was performed using specimens adhered to glass coverslips. For quantitative analysis of NET presence, ≥ 4 coverslips per subject (in most cases 6) were reacted with rabbit anti-human NE IgG (ab21595, Abcam, Cambridge, UK; 1:50) using propidium iodide (PI) (P4170, Sigma Aldrich, Schnelldorf, Germany) for DNA counterstaining. Additional specimens from many of the subjects (from all that provided more than 4 usable coverslips) were used for the parallel documentation of NET and neutrophil morphology. These specimens were either stained for citH3 (rabbit anti-human CitH3 (citrullin 2 + 8 + 17) IgG, Abcam ab77164; 1:50–1:100) using PI as DNA stain, or double-stained for citH3 and PAD4 (mouse monoclonal anti-PAD4, (Abcam ab128086; 1:100), with DAPI (Sigma-Aldrich, Germany) as DNA stain. DNase treated control specimens were stained for NE. DyLight®-conjugated goat anti-rabbit IgG (Abcam ab96883; 1:100) and TRITC-labelled goat anti-mouse IgG (Abcam ab 6786; 1:100) were used as secondary antibodies. Analyses were done in a Zeiss LSM 510 meta UV CLSM (Carl Zeiss GmbH, Vienna, Austria).

### Quantitative assessment of NET and neutrophil abundance

Specimens stained with anti-NE and PI (n ≥ 3 per tested subject, see above) were used to evaluate the presence of NETs and neutrophils according to four categories: non-activated (morphologically undisturbed) neutrophils, activated/NET-forming neutrophils, large amounts of NETs, minor traces of NETs. All categories were assessed in a dichotomous manner (applies or does not apply) according to pre-defined inclusion criteria (Table 2). NETs and NET-forming neutrophils were identified according to the typology provided by the key literature to date [9, 14, 27], and by comparison with NETs generated *in vitro* from human neutrophils in our own lab [24]. NET-forming neutrophils were identified primarily according to their changed nuclear morphology (Table 2). Choice of this criterion is based on the observation that loss of the characteristic lobular form of the nucleus is a recognisable and reliable initial morphological alteration in the NETosis process, when the morphology of cytoplasm and organelles is still intact [9]. The presence of NE in the nucleus was not used as a criterion to identify NET-forming neutrophils because this feature is not mandatory in the early phase of the NETosis process [24, 28].

**Table 2:** Criteria for assessment of neutrophils and NETs in categories

Category	Predefined criteria			
Non-activated neutrophil	intact cell	lobulated nucleus	positive NE staining in cytoplasmic granulae only	
Activated/NET-forming neutrophil	intact cell	altered nuclear morphology	positive NE staining in cytoplasm and/or nucleus	
Large amounts of NETs	extracellular fibrous structures	positive PI-staining	positive NE staining	extended, confluent and/or overlapping formations occupying at least 1 mm^2^
Minor traces of NETs	extracellular fibrous structures	positive PI-staining	positive NE staining	≤10 small-sized (≤50 μm^2^) nonoverlapping items per 100 mm^2^

Classification into a category requires fulfillment of all criteria listed for that category. Note that an individual sputum sample (and all CLSM specimens prepared from it) could apply to both neutrophil categories (‘non-activated’ and ‘activated/NET-forming’). By contrast, an individual sputum sample could not apply to both NET-related categories because a sample was assigned to the ‘large amounts’ category when only one CLSM specimen fulfilled the criteria for this category, irrespective of whether any other specimen from the same sample fulfilled only the criteria for ‘minor traces’, or was free of NETs

Categories of NET abundance were classified as follows: (i) ≥ 10 small-sized (≥50 μm) non-overlapping items per 100 mm^2^ coverslip surface area, mainly associated with individual neutrophils, were defined as ‘minor traces’; (ii) extended, confluent and/or overlapping formations with numerous neutrophils occupying at least about 1 mm^2^ (in nearly all cases more than one quarter of the coverslip surface) were defined as ‘large amounts’. A patient was assigned to the large amounts group when at least one CLSM specimen fulfilled the criteria for “large amounts”, irrespective of whether any other specimen of the same patient exhibited only minor traces. Results are presented as percentages of total individuals sampled per study group (Fig. 3a) and per COPD severity stage (Fig. 3b). This approach was chosen as a feasible alternative to methods of numerical assessment since testing to adapt fluorometry-based techniques of NET quantification applicable to *in vitro* use (cf./eg. [29, 30]) had failed to provide reliable results with sputa of highly heterogeneous consistency.

### Morphometry-based quantification of NETs

The images of each sample used for abundance assessment were also employed to provide a morphometry-based estimation of NET quantity. The outlines of areas covered by NET DNA were traced on red-channel (PI) CLSM photographs and binary images of these areas were generated using Adobe Photoshop. Non-NET DNA (mainly intact or disintegrating nuclei of neutrophils) was excluded. NET areas were measured with the particle analysis tool of the software ImageJ, and NET area ratios were calculated for each sample (Figs. 3c, d).

### Electron microscopy

Scanning electron microscopy (SEM) served for routine control of NET presence and was performed on specimens on coverslips from most of the subjects (from all that provided produce more than 4 usable specimens of this kind). These were dehydrated in ethanols, critical-point-dried with liquid CO_2_, sputter-coated with gold, and analysed in a ESEM XL30 (FEI Company, PHILIPS, Eindhoven, Netherlands).

Transmission electron microscopy (TEM) was used in two ways: (i) For the ultrastructural localisation of NE and CitH3 within the NETs. This was done by immunoelectron microscopy of sputa on gold grids. Primary antibodies were the same as described for CLSM analysis above, 5 nm colloidal gold conjugated goat anti-rabbit IgG (Abcam ab27235, 1:10) served as secondary antibody. Immunolabelled specimens were negative-stained with uranyl acetate (1 % aqueous solution, 1 min on ice), and (ii) for analysis of NETs and sputum components on ultrathin sections of specimens collected on Aclar® strips. These specimens were dehydrated in a series of ethanols, infiltrated with Glycidether 100 (Serva) epoxy resin via propylene oxide, and polymerised at 60 °C. After removal of the Aclar® plastic, ultrathin sections (80 nm) were cut on a Leica Ultracut 7 microtome, post-stained with 0.5 % uranyl acetate and 3 % lead citrate, and – as with the immunogold specimens – viewed in a LEO EM 910. Digital images were made with a *Sharp: Eye* camera system (Troendle, Moorenweis, Germany). Specimens used for analysis were selected from those that showed abundant NET presence in the immunostaining results.

### Statistical methods

Power calculations undertaken prior to subject recruitment using the statistics software R showed that, assuming a difference of at least 50 % in the presence of NETs between patient groups and control groups, sample sizes of 14 or higher would be adequate to detect intergroup differences with 80 % power at the 5 % significance level (α = 0.05).

Statistical analyses were carried out using the R package npmv (R Core 2014) [31, 32] which performs nonparametric global multivariate analysis of variance (MANOVA) tests. In addition to these global hypothesis tests, the package executes a closed multiple testing procedure identifying significant differences between groups [33]. This method allows for non-normal data and ordinal scales, as provided by the results of the present study. Eight cases with missing values were deleted (complete case analysis). In order to ensure that the younger smoking controls (Table 1) did not introduce bias in the inference, analyses were carried out with and without this group (Table 3). A multiple testing procedure controlling the maximum overall type 1 error (also implemented in the R package npmv) was performed to test for significant differences between study groups (exacerbated COPD/hospitalised, stable COPD, smoking controls, non-smoking controls) and between COPD severity groups (GOLD grades 3/4, GOLD grades 1/2, smoking controls, non-smoking controls) regarding the following variables: presence of NETs (in minor traces or large amounts), non-activated neutrophils, activated/NET-forming neutrophils (all ordinal), and percentage of NET coverage in sputum preparations and FEV1 (both continuous). Nonparametric ‘relative effects’ are provided as effect estimators. The ‘relative effects’ give an indication of stochastic superiority, i.e. they measure the probability that a value obtained from one experimental group is larger than a value randomly chosen from the whole trial including the controls. This statistical approach has been found suitable for studies aiming to demonstrate effects that are both statistically significant and clinically relevant [34, 35]. Spearman’s rank correlation coefficient was used to assess the degree of monotonic relationship between the variables FEV1 and percentage of NET coverage.

**Table 3:** Estimated nonparametric relative effects showing intergroup differences as probabilities

	Smoking controls omitted					Smoking controls included				
	FEV1	Non-activated neutrophils	NET-forming neutrophils	NETs	NET covered area	FEV1	Non-activated neutrophils	NET-forming neutrophils	NETs	NET covered area
Exacerbated COPD	0.27908	0.52941	0.56471	0.72680	0.75098	0.20931	0.53676	0.56225	0.75686	0.79363
Stable COPD	0.41690	0.45798	0.50280	0.49020	0.47479	0.31968	0.46534	0.50035	0.53081	0.52276
Smoking controls						0.76254	0.47794	0.50735	0.38408	0.36808
non-smoking controls	0.83725	0.52941	0.43137	0.28693	0.28431	0.74559	0.53676	0.42892	0.33137	0.32402
COPD grades 3/4	0.27669	0.51089	0.56100	0.64670	0.64089	0.20752	0.51825	0.55855	0.68192	0.68818
COPD grades 1/2	0.60784	0.41830	0.43137	0.41503	0.43682	0.47222	0.42565	0.42892	0.45425	0.47794
Smokong controls						0.76254	0.47794	0.50735	0.38408	0.36808
Non-smoking controls	0.83725	0.52941	0.43137	0.28693	0.28431	0.74559	0.53676	0.42892	0.33137	0.32402

Evaluation of study data with a closed testing procedure performing multiple hypothesis tests simultaneously, significant endpoints in bold. Summary of estimated nonparametric relative effects of tested variables with and without inclusion of the smoking controls. Estimated relative effects measure the probability that a value obtained from one experimental group is larger than a value randomly chosen from the whole trial including the controls. The procedure yields similar conclusions about the significance of differences between study groups and between COPD severity groups, irrespective of whether smoking controls are excluded or included

## Results

**Fig. 1:**
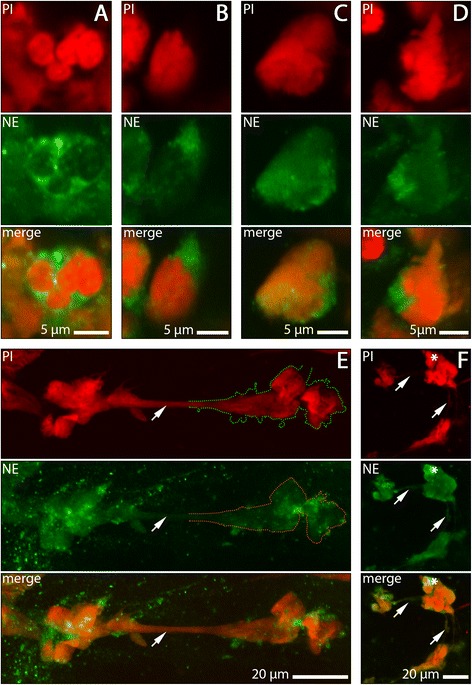
NET-forming neutrophils and NETs in sputum samples of COPD subjects stained with anti-NE (green) and PI (red). **a** non-activated neutrophil with lobulated nucleus and cytoplasmic localisation of NE. (B-D) activated neutrophils at different stages of NET formation. **b** nucleus swollen, NE staining still confined to cytoplasm. **c** NE present in both, cytoplasm and nucleus. **d** ruptured cell in early phase of NET release. **e**, **f** Representative examples of NET morphology. NETs are characterised by extensive colocalisation of DNA and NE (the lack of absolute DNA-NE overlap is explainable by the irregular molecular structure of NETs [24], and by secondary alteration this structure during sputum transport). **e** Long stretch of NET-DNA (arrow) extending between two dense aggregates. In part of the motif, overlap of DNA stain and NE stain is illustrated by stippled lines. **f** Clusters of NET-DNA, cell debris and an intact neutrophil (asterisk) connected by thin NET trajectories (arrows)

**Fig. 2:**
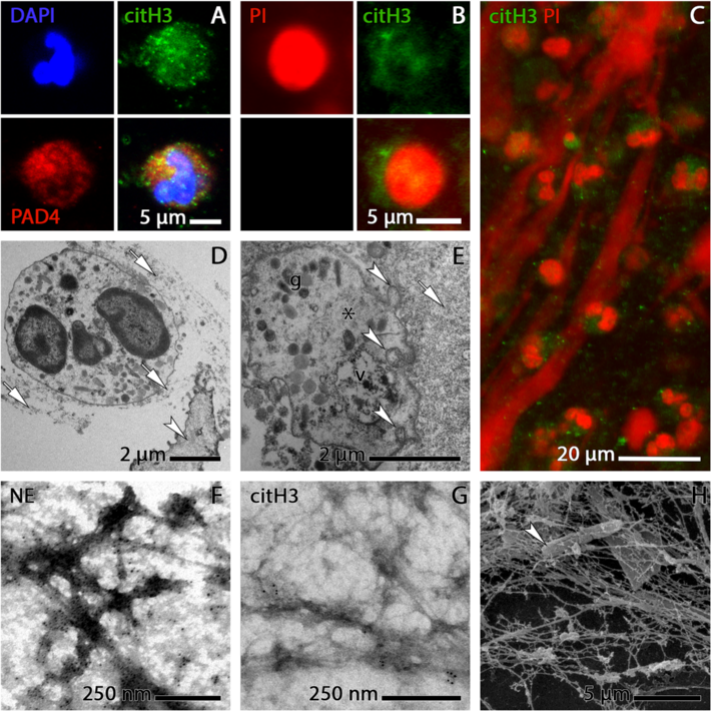
Identification of NET-forming neutrophils and NETs in COPD sputum by additional methods of analysis. **a-c** CLSM images. **a** activated/NET-forming neutrophil stained for citH3 (green) and PAD4 (red), DNA blue. **b** activated/NET-forming neutrophil stained for citH3 (green) and DNA (red). **c** Overview image of citH3-stained specimen showing large trajectories of NET DNA intermingled with numerous activated/NET-forming and non-activated neutrophils. The presence of citH3 and PAD4 in both the cytoplasm and the nuclei of the neutrophils conforms with the seminal study on histone deimination in NETosis by Neeli et al. [24, 68] and with our own previous fndings on NET micromorphology [24, 68]. **d-e** TEM images of ultrathin sections. **d** Tight attachment of NETs (arrows) to the surface of a bronchiolar epithelial cell (arrowhead) from COPD sputum; NET fibres are also wrapped around an apparently intact (non-NET-forming) neutrophil. **e** Tangential section through an activated/NET-forming neutrophil outside the nuclear region. The cell is embedded in a mass of NETs clotted with amorphous sputum substance (arrow) and contains various granulae (g), a presumably autophagic vacuole (v), indication of vesicular traffic (arrowheads), and NET-like fibres (asterisk). **f-g** TEM images of on-grid immunogold stained sputum NETs. **f** NE epitopes are abundant in the aggregations of organic matter along the NET fibres. **g** Labelling for citH3 is far less abundant than NE stain and clustered at distinct sites of the NET meshwork. **h** SEM image of sputum NETs with an entangled bacterium (arrowhead)

The morphological analyses using CLSM, TEM and SEM show clearly that sputa of both patients with acutely exacerbated COPD and patients with stable COPD are characterised by a massive presence of NETs and neutrophils at various stages of NET formation (Figs. 1 and 2).

**Fig. 3:**
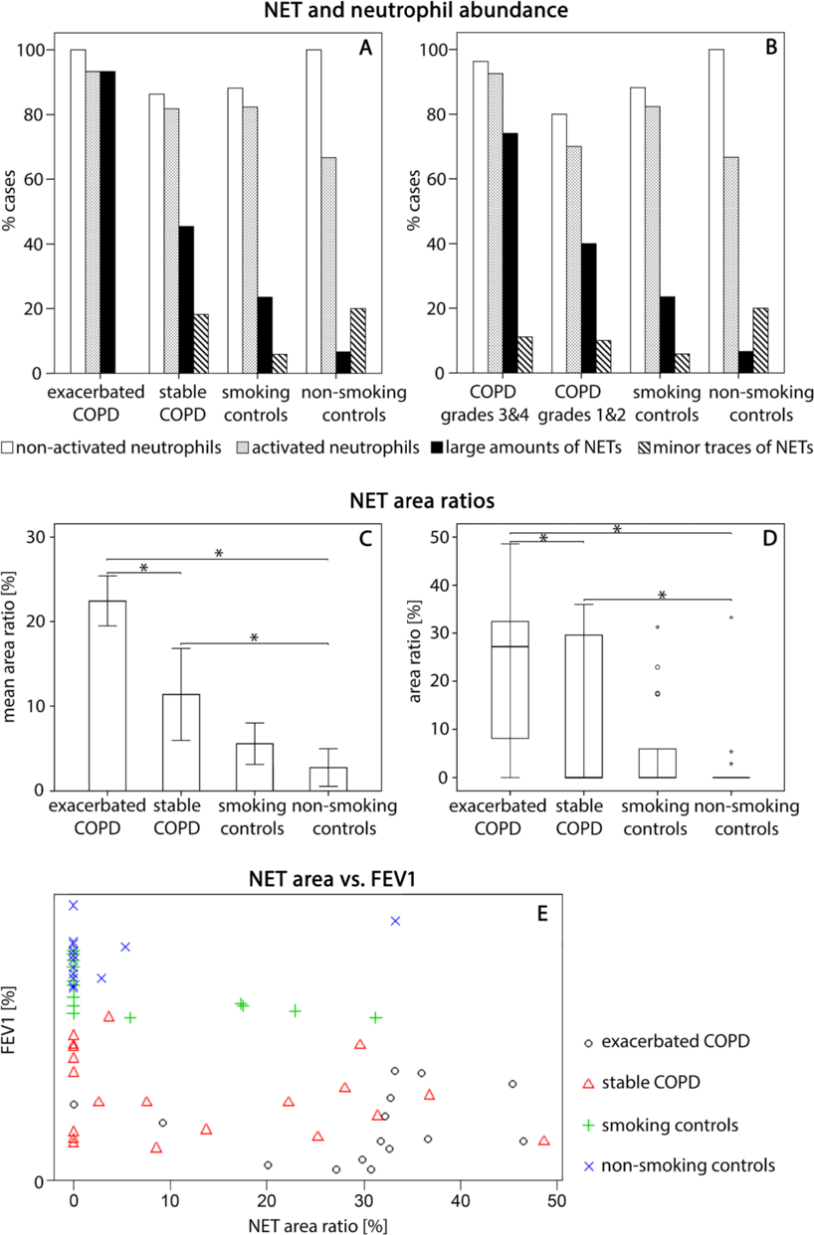
Quantification of neutrophils and NETs. **a, b** Evaluation in categories, bars represent percentages of total individuals sampled per study group (**a**) and per COPD severity stage (**b**). **c, d** Percentages of NET coverage in sputum preparations. **c** Bar chart showing means ± standard errors. **d** Boxplots with medians and interquartile ranges, whiskers have maximum 1.5 interquartile range. Supercript asterisks indicate significant differences between groups (*P* < 0.05). **e** Scatter plot illustrating the relation between FEV1 and the percentage of NET coverage, study groups represented by different symbols (Spearman’s rank correlation coefficient *ρ* = −0.562, *P* < 0.001)

Quantitative evaluation in categories (Fig. 3a, b) provides more detailed information on this finding. It shows that sputum samples from exacerbated COPD are most seriously affected. They contain large amounts of NETs and NET-forming neutrophils in more than 90 % of patients. The sputa of subjects with stable COPD, and remarkably also those of the smoking controls without airflow limitation, contain large amounts of NETs in about 45 and 25 % of the patients sampled, respectively, and NET-forming neutrophils occur in about 80 % of each of these groups (Fig. 3a). By contrast, the sputa from non-smoking controls were, with the exception of one subject, either completely devoid of NETs or contained them only in minor traces (Fig. 3b). The high content of NETs in one of the control samples was most likely caused by an asymptomatic respiratory infection (the subject exhibited no characteristic clinical symptoms at the time of sampling).

These results further demonstrate that the abundance of NET in the sputa of COPD subjects correlates with the severity of airflow limitation. The sputum structure of most subjects with very severe and severe COPD (GOLD grades 4 and 3, respectively) is dominated by extended aggregates of fully spread NETs, associated with neutrophils in various stages of NETosis. The presence of spread NETs is also characteristic for the sputa of subjects with moderate and mild COPD (GOLD grades 2 and 1, respectively) but with a clearly lower prevalence as compared to grades 4 and 3. This holds also for the presence of NET-forming neutrophils (Fig. 3b). However, the absence of airflow limitation in the controls was only rarely accompanied by a full absence of NETs. Indeed, quite to the contrary, spread NETs and NET-forming neutrophils were also characteristic of many of the control sputa, although with a clear difference between smoking and non-smoking controls. A considerable proportion of the sputa of smoking controls contained NETs, in some cases even in large amounts. By contrast, the sputa of non-smoking controls were (with one exception) largely free of these contents (Fig. 3a, b).

The morphometric estimation of NET content via measurement of NET-covered area in the sputum preparations further substantiates the findings of the category-based evaluation. Percentages of NET coverage are the highest in patients with exacerbated COPD, those of patients with stable disease are less, followed by those of smoking controls and non-smoking controls (Fig. 3c, d). There is a significant negative association between the percentage of NET coverage and FEV1, the measure of airflow rate (Spearman’s rank correlation coefficient ρ = −0.562, *p* < 0.00001) (Fig. 3e).

Omitting the smoking controls, global hypothesis tests followed by a closed testing procedure show highly significant differences between the three study groups in the variables ‘presence of NETs’ (in large amounts or minor traces), ‘percentage of NET-covered area’, and ‘FEV1’ (overall type 1 error rate α < 0.001). Based on this finding, we identified these three variables as significant endpoints (i.e. as variables that can be usefully employed to differentiate between the study groups). Exemplified for FEV1 (the main classification criterion of COPD), the estimated nonparametric relative effect for healthy controls (i.e. the estimated probability that a randomly selected healthy control has a larger FEV1 than a randomly selected person from the whole trial including the healthy controls) is 83.7 %. The corresponding probability estimate for exacerbated COPD patients is only 27.9 % (confirming the simple fact that normal lung function has a rather low probability of occurrence in exacerbated COPD). Estimated nonparametric effects for ‘presence of NETs’ and ‘percentage of NET-covered area’, are 72.7 and 75.1 %, respectively, for exacerbated COPD, 49.0 and 47.5 %, respectively, for stable COPD, but only 28.7 and 28.4 %, respectively, for non-smoking controls (Table 3). Thus, COPD patients with exacerbations have clearly the highest probability of NETs occurring in their sputa, distantly followed by patients with stable COPD, irrespective of whether NETs are assessed in ordinal/dichotomous format or by morphometry on the sputum preparations.

Similar to these evaluations, comparing non-smoking controls with COPD subjects sorted by COPD severity groups (GOLD grades 3/4 and 1/2) by the same testing procedure as before, the variables ‘presence of NETs’, ‘percentage of NET-covered area’ and ‘FEV1’ are identified as significant endpoints (overall type 1 error rate α = 0.008).

Estimated probabilities yielded from analyses that included the smoking controls diverge from those obtained omitting these controls, but still allow similar conclusions about the significance of intergroup differences and endpoints (Table 3).

## Discussion

The present study has shown that sputa of patients with all grades of COPD, exacerbated or stable, are characterised by the presence of large amounts of NETs and NET-forming neutrophils (Fig. *3*a, b). This provides an important extension of our previous detection of NETs in exacerbated COPD [24] and supports the assumption that NETs also contribute to chronic inflammation in COPD, as previously documented for a variety of other diseases, including those of the respiratory system [17, 19, 20]. In view of the high prevalence of COPD and its associated social burden, our findings clearly highlight the need for further research into the role of NETosis in COPD pathogenesis, particularly in relation to the development of new diagnostic tools and treatment strategies. Developing a standardised procedure of NET assessment could provide an easily applicable and reliable means to monitor the extent of inflammation and disease progression. Our results also add further weight to considerations of manipulating NETosis for therapeutic purposes. Intervention by local (non-systemic) application of antagonists to neutrophil IL-8 receptors has already been identified as a promising approach to reduce tissue damage by neutrophils in pulmonary disease, including CF and COPD [36, 37]. This may now be taken further and extended to other molecular targets (eg. inhibitors of MPO and PAD4, and ROS scavengers) that have been recently defined as possible therapeutics to repress NET formation [17].

### Stage dependence of COPD-associated NETosis

The diagnostic and therapeutic relevance of our results is further strengthened by the finding that COPD related NETosis clearly correlates with the impairment of lung function, which is considered one of the best possible indicators of disease severity. All features of NETosis are more frequently seen in the sputa of patients with exacerbations and at GOLD grades 3 and 4 than in those of patients with stable COPD and at GOLD grades 1 and 2 (Fig. 3b). Together with the accumulating evidence that NETs are potent inductors of cell and tissue damage in inflammatory disease (eg. [15–17, 38]), these results provide initial evidence that NETosis is a harmful factor rather than a simple epiphenomenon of COPD. This conclusion is in agreement with most recent work on sputum NETs in CF [39], but it will certainly require time-series studies that follow a particular cohort of patients to obtain further verification and to examine progression over time.

NET forming neutrophils, in contrast to apoptotic cells, were found to lack signals inducing their clearance by phagocytes [17]. NETosis is able to function in self-perpetuating cycles, driving the process into a detrimental excess (eg. [40]), particularly in situations of non-microbial induced (sterile) inflammation (eg. [25, 41, 42]). The massive presence of NETs in the sputa of COPD patients (Figs. 1*,*2 and 3) may indicate exactly such an over-reactive response beyond the requirements of anti-microbial defence. This interpretation is consistent with the experience from patients with CF [19], and with the conclusion on adverse NET effects in a variety of other inflammatory lung diseases in the recent literature [43]. It is also in agreement with recent *in vitro* evidence of variants of the NETosis mechanism that are faster than the standard type. These mechanisms are activated in response to bacterial challenge and may be most appropriate for a role in lung defence. This holds particularly for a mechanism mediated by immunoglobulin A, the predominant antibody to provide mucosal protection against pathogens [44], but also for a second mechanism that leaves neutrophils after NET extrusion viable and phagocytotically active [29]. In the light of the present results it would be important to test whether these NETosis mechanisms are active in COPD.

Our observation that NETs are present in patients with exacerbated COPD even though they were treated with systemic corticosteroids is in agreement with recent experimental evidence that such medication is insufficient to reduce NET formation [25, 26]. Furthermore, detrimental effects of NETs in COPD lungs may not be confined to direct damage to the epithelia [45], but may also include indirect damage via promotion of autoimmune reactions against NET components (cf. [46]). Together, this could be a relevant contribution to the fatal spiral of decline in lung function that characterises the progression of COPD. Self-perpetuating NETosis may also help to explain why COPD subjects exhibit persistent airway inflammation and even increased numbers of sputum neutrophils after one year of cessation of smoking [21]. However, COPD has been recently described as a heterogeneous disease with a non-uniform lung function decline [47]. It therefore remains to be established whether individual disposition to NETosis is relevant to understand the different COPD phenotypes.

### Implication of COPD-associated NETosis for tumour development

NETosis is an important mechanism to enhance oxidative stress, not least through the abundant release of myeloperoxidase (MPO) [28, 48], a potent generator of potentially tissue harming oxidants [49–51]. In view of the well established close relationship between oxidative stress, chronic inflammation, and cancer (e.g. [52–54]), it would appear worthwhile to explore whether routine measurement of sputum NETs could aid tumour risk assessment and tumour prevention in COPD.

### Smoking and non-smoking controls

Further indication that NETosis may be a potentially harmful factor in the development of COPD is provided by the finding that NETs are not only abundant in the sputa of subjects with COPD, but also in those of a proportion of smokers who do not exhibit airflow limitation (Fig. 3a-d). It is well established that in westernised countries, smokers constitute the main candidate population to develop COPD [47, 55]. The results of the present work could indicate that cigarette smoke-induced NETosis commits some smokers into a self-perpetuating cycle of inflammation and respiratory damage, eventually terminating in full scale COPD. Known variation among smokers in their risks of developing COPD (average about 50 %) is well documented [56, 57] and may be directly reflected in the high presence of NETs in the sputa of some of the smoking controls (Fig. 3). A pathological relevance of the high presence of NETs in the sputa of the smokers is supported by the fact that the difference between the smokers and the stable COPD group is not pronounced (Fig. 3c, d). This has alarming implications just because the smokers group is on average younger: previous work demonstrates that neutrophil recruitment in the lung, the key requisite of NET formation, increases with age, even in healthy subjects [58]. A negative influence of NET presence in the lungs of smokers is also supported by our observations when searching for participants in the study. Not only that we could not find smokers over 60 with sufficient lung function, but even some of those screened that were clearly younger (at around 40) had a considerable impairment and were thus diagnosed with COPD for the first time.

All this is in agreement with the accumulating evidence that commitment to NETosis depends on the interplay between the inducing external factors and the intrinsic genetic framework of the individual host [59]. This interplay may also aid in understanding the rather surprising presence of NET traces in about 20 % of the samples from non-smoking controls, and the presence of activated/NET-forming neutrophils in almost 70 % of these control samples (Figs. 2 and 3). Whether this indicates a short term low-level (‘routine’) employment of NETosis in pulmonary antimicrobial defence, or involves a side effect of sputum induction (see below), remains to be tested. Neutrophil recruitment into alveolar spaces may be regarded as an established contributor to normal phagocytotic clearance of inhaled bacteria and organic dust (eg. [60, 61]). The underlying molecular regulation and interplay with macrophages has been clarified over recent years [62–64].

### Possible bias from sputum induction

On the basis of the present literature, it cannot be entirely ruled out that sputum induction according to protocol of the ECLIPSE study [8] leads to the formation of additional NETs. Previous work has found that sputum induction can evoke a short-lived neutrophilic response and may also exert prolonged inflammatory stimuli, especially when repeatedly applied (e.g. [65]). However, there is also evidence that the effect is not excessive, even in patients with COPD [66] and influences, within the time required for sputum acquisition, neutrophil content rather than NET content. The ECLIPSE study itself mentions that sputa from healthy airways induced by this method contain intact neutrophils. This is in agreement with our results from non-smoking controls (Fig. 3a, b). The low presence of NETs in these control sputa, on the other hand, supports the view that the effect of sputum induction on the NET content of the harvested sputa is limited. This appears even more plausible as it takes clearly more than one hour until activated neutrophils are ready to release NETs [67]. The extreme difference in NET content that is apparent between these controls and the sputa of the COPD subjects (Fig. 3a-c) suggests that the mass of NETs found in the latter results from the disease and not from sputum induction.

## Conclusions

From the results of this work we conclude that (i) NETs are the dominant (if not exclusive) source of sputum DNA in COPD, (ii) In view of the accumulating evidence of NET-mediated tissue damage from a variety of chronic inflammatory diseases including such of the lung (eg. [43]), NETs could contribute to lung function decline in a high proportion of the patients with COPD, (iii) this detrimental development may already be initiated in smokers without airflow limitation, (iv) NET recruitment at very low levels is a component of normal lung defence, and that (v) routine assessment of the NET burden could help to optimise the effective use of medication (e.g. the application of steroids [22, 23]), and tumour risk assessment and tumour prevention in COPD.

## Abbreviations

ATS/ERS: American Thoracic Society/European Respiratory Society
CF: Cystic fibrosis
citH3: Citrullinated histone 3
CLSM: Confocal laser scanning microscopy
COPD: Chronic obstructive pulmonary disease
DAPI: 4′,6-diamidino-2-phenylindole
DTT: Dithiothreitol
FEV: Forced expiratory volume per second
IL-8: Interleukine 8
MPO: Myeloperoxidase
NADPH: Nicotinamide adenine dinucleotide hydride
NE: Neutrophil elastase
NETs: Neutrophil extracellular traps
NETosis: Distinct type of neutrophil cell death associated with the release of NETs
PAD4: Peptidyl arginine deiminase 4
PI: Propidium iodide
ROS: Reactive oxygen species
SEM: Scanning electron microscopy
TEM: Transmission electron microscopy
TRITC: Tetramethylrhodamine isothiocyanate
